# Amino Acid Trafficking and Skeletal Muscle Protein Synthesis: A Case of Supply and Demand

**DOI:** 10.3389/fcell.2021.656604

**Published:** 2021-05-31

**Authors:** James P. White

**Affiliations:** ^1^Department of Medicine, Duke University School of Medicine, Durham, NC, United States; ^2^Duke Molecular Physiology Institute, Duke University School of Medicine, Durham, NC, United States; ^3^Duke Center for the Study of Aging and Human Development, Duke University School of Medicine, Durham, NC, United States

**Keywords:** skeletal muscle, branch chain amino acids, protein synthesis, BCKD, branched-chain α-ketoacid dehydrogenase, AMPK (5′-AMP activated kinase), mammalian target of rapamycin

## Abstract

Skeletal muscle protein synthesis is a highly complex process, influenced by nutritional status, mechanical stimuli, repair programs, hormones, and growth factors. The molecular aspects of protein synthesis are centered around the mTORC1 complex. However, the intricacies of mTORC1 regulation, both up and downstream, have expanded overtime. Moreover, the plastic nature of skeletal muscle makes it a unique tissue, having to coordinate between temporal changes in myofiber metabolism and hypertrophy/atrophy stimuli within a tissue with considerable protein content. Skeletal muscle manages the push and pull between anabolic and catabolic pathways through key regulatory proteins to promote energy production in times of nutrient deprivation or activate anabolic pathways in times of nutrient availability and anabolic stimuli. Branched-chain amino acids (BCAAs) can be used for both energy production and signaling to induce protein synthesis. The metabolism of BCAAs occur in tandem with energetic and anabolic processes, converging at several points along their respective pathways. The fate of intramuscular BCAAs adds another layer of regulation, which has consequences to promote or inhibit muscle fiber protein anabolism. This review will outline the general mechanisms of muscle protein synthesis and describe how metabolic pathways can regulate this process. Lastly, we will discuss how BCAA availability and demand coordinate with synthesis mechanisms and identify key factors involved in intramuscular BCAA trafficking.

## Brief Overview of Protein Translation

**FIGURE 1:**
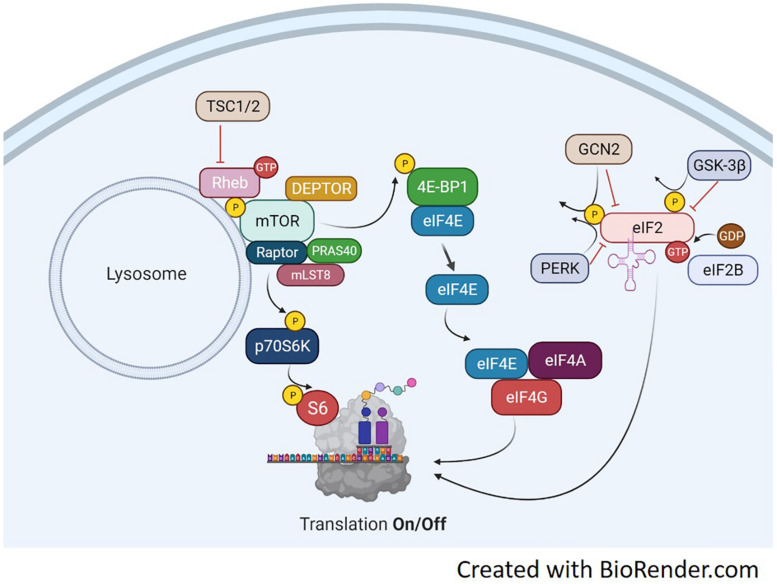
Translation Overview. Protein translation occurs, in part, by mTORC1-dependent regulation of p70S6K and the 4E-BP1/eIF4E complex and the mTORC1-independent eIF2 complex. mTORC1 activation is achieved through several points of regulation including, but not limited to phosphorylation at ser2448, inactivation of the TSC1/2 complex, binding of GTP-bound RHEB and translocation to the lysosome. Upon activation, mTORC1 will phosphorylate p70S6K at Thr389. This activates p70S6K to phosphorylate ribosomal protein S6 at ser236/236 and initiate RNA translation. mTORC1 can also regulate the eIF4E complex by phosphorylating 4E-BP1 which results in detachment of 4E-BP1 from eIF4E. This frees eIF4E to bind with eIF4A and eIF4G which will initiate binding of mRNA to the ribosome. A third point of translation initiation is the eIF2 complex. eIF2 forms a ternary complex with GTP and the Met-rRNA binds to the 40S ribosomal subunit to start the translation process. Its regulation is mediated through phosphorylation by various kinases including GSK-3β, PERK, and GCN2. Phosphorylation of eIF2 inhibits the guanine nucleotide exchange function of eIF2B and prevents translation.

Protein synthesis is regulated primarily at the initiation phase of protein translation. A series of signaling proteins, referred to as eukaryotic initiation factors (eIFs), ultimately control this process and depend on upstream signals to modulate their activity. The pathways involved in protein synthesis are extensive, however, two different events govern the translation process, described in Figure 1. The binding of the methionyl tRNA (met-tRNA) to the 40S ribosomal subunit is regulated by the eukaryotic initiation factor 2 eIF2 (Price and Proud, 1994). eIF2 binds GTP and the eIF2-GTP-met-tRNA binds to the 40S ribosomal complex forming the 43S preinitiation complex. Once the start codon of an mRNA binds to the complex, GTP is hydrolyzed back to GDP. The complex cannot form the 43S preinitiation complex until GTP is reformed (Panniers and Henshaw, 1983; Price and Proud, 1994). The enzyme guanine nucleotide exchange factor eIF-2B will return GDP back to GTP and allow the complex to be active again. The regulation of this process is at the phosphorylation state of the α subunit of eIF2. When eIF2 is phosphorylated, eIF2B is inhibited from recycling GDP back to GTP and translation is stopped (Rowlands et al., 1988). eIF2 is phosphorylated by several kinases including double stranded RNA-dependent protein kinase (PKR), heme-regulated inhibitor kinase (HRI), eukaryotic translation initiation factor 2-alpha kinase 3 (PERK), the yeast general control non-derepressible 2 (GCN2) (Proud, 2005), and more recently, glycogen synthase kinase-3 beta (GSK3β) (Welsh et al., 1998). Each kinase appears to target eIF2 under different cellular stresses. PKR is activated in the presence of double stranded RNA (dsRNA) commonly found from viral infections (Meurs et al., 1990). PKR inactivates eIF2 as a protective mechanism to shut down protein synthesis and stop viral replication. HRI was discovered in reticulocytes (Crosby et al., 2000). When heme is deprived from reticulocytes protein synthesis is shut off. This process is associated with the inactivation and subsequent phosphorylation of eIF2. Of these regulators, only GCN2, GSK3β, and PERK regulate eIF2 based on amino acid availability. Their respective actions are illustrated in Figure 1. GCN2 is extensively studied in yeast during inhibition of protein synthesis by amino acid deprivation (Marton et al., 1993). It inactivates eIF2 by phosphorylation at serine 51. GSK3β has been shown to be a key regulator in insulin-dependent protein synthesis in skeletal muscle by inactivating eIF2 through phosphorylation at serine 540 (Jefferson et al., 1999). GSK3β and its role in muscle protein synthesis will be discussed in more detail later in the review. PERK has been found to target and inhibit eIF2 by phosphorylation at serine 51 under various conditions, including iron or heme-deficiency, amino acid starvation, viral infection, and accumulation of unfolded proteins (Dever, 2002).

The remaining regulatory mechanisms of protein translation are downstream of the mammalian target of rapamycin complex 1 (mTORC1). mTOR is a Ser/Thr kinase involved in a variety of processes including cell growth and differentiation, protein synthesis, and actin cytoskeletal organization. The mTORC1 complex can phosphorylate both 4E-BP1 and p70S6K to activate two downstream translation pathways, seen in Figure 1. Upon phosphorylation, the 4E-BP1 detaches from eIF4E and binds the eIF4F complex (Mader et al., 1995). The eIF4F complex recruits the 40S ribosomal subunit to mRNA through the 5′-cap structure (Proud, 2007). The eIF4F complex consists of three subunits each with distinct functions. eIF4E binds the 5′mRNA, eIF4A is an ATP-dependent RNA helicase and eIF4G serves as structural support for both eIF4E and 4A to form the eIF4F complex. eIF4E has been identified as a main regulatory protein for translation initiation through the eIF4F pathway. During instances of hypophosphorylation, 4E-BP1 remains bound to eIF4E and translation is turned off (Mader et al., 1995).

## The Canonical IGF/Akt/mTORC1 Pathway

**FIGURE 2:**
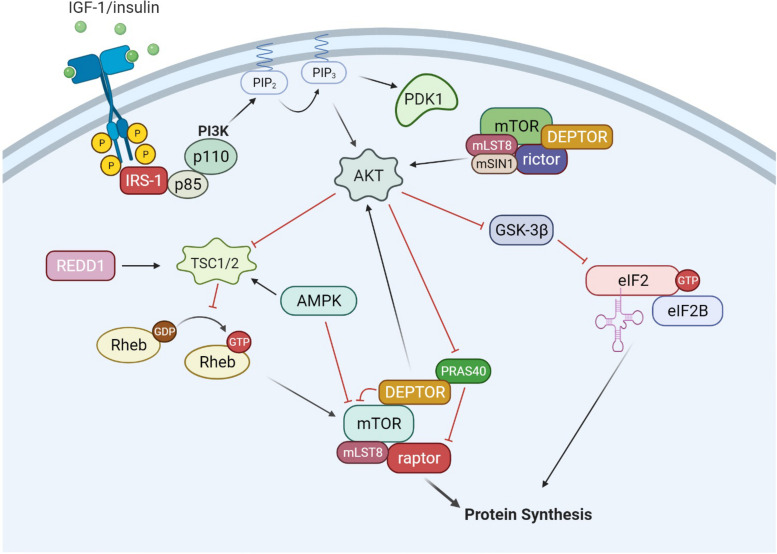
IGF-1/Akt signaling. The binding of growth factors such as IGF-1 and insulin activates receptor tyrosine kinases (RTKs), which recruits and activates IRS-1. IRS-1 then activates phosphatidylinositol-4,5-bisphosphate 3-kinase (PI3K), which consists of a regulator subunit (p85) and a catalytic subunit (p110). PI3K generates phosphoinositide 3,4, 5-phosphate (PIP_3_). PIP3 recruits and activates PDK1 and Akt. In addition to PIP3 signaling, mTORC2 can also phosphorylate and activate Akt. From there, Akt can signal through both mTORC1 and eIF2 pathways to increase protein synthesis. Akt can phosphorylate and inactivate GSK-3β, which is an inhibitor of the eIF2 complex. Akt can also activate the mTORC1 complex through inactivation of TSC1/2 and PRAS40. AMPK is a negative regulator of the IGF-1/Akt pathway, inhibiting mTORC1 activity through activation of the TSC1/2 complex and direct inhibition of mTORC1.

The upstream pathways controlling mRNA translation are, in part, through the IGF-1/mTORC1 pathway (Glass, 2010), illustrated in Figure 2. The IGF-Akt (PKB) signaling pathway is well established for its role in regulation of skeletal muscle mass controlling both protein synthesis, degradation, and apoptotic pathways (Frost and Lang, 2007). The binding of IGF-1 activates the receptor tyrosine kinase IGF-1 receptor and recruits insulin receptor substrate (IRS), in particular IRS-1 (Sun et al., 1991; Yamauchi et al., 1998). This leads to the activation of phosphatidylinositol 3′-kinase (PI3K) and the eventual activation of the serine-threonine kinase Akt (PKB) via phosphorylation at serine 473 (Alessi et al., 1997; Andjelkovic et al., 1997; Moelling et al., 2002). Akt is a focal point in insulin and IGF-1 signaling in a variety of tissues. Akt is also phosphorylated by mTORC2 (Sarbassov et al., 2005), which will be discussed later in the review. Upon activation, Akt is involved in a multitude of downstream pathways that will promote muscle growth. During skeletal muscle hypertrophy, Akt activation is increased when examined *in vivo* (Bodine et al., 2001) and in cultured myotubes (Rommel et al., 2001). In addition, a genetically altered, constitutively active Akt was able to induce muscle hypertrophy independent of additional treatments (Bodine et al., 2001; Lai et al., 2004). Akt acts through mTORC1 pathways to initiate and enhance protein synthesis. In addition, Akt can enhance protein synthesis through inhibition of proteins that impede protein synthesis such as GSK-3β and PRAS40. As mentioned previously, GSK3β is an inhibitor of protein synthesis through phosphorylation and inhibition of eIF2. Akt phosphorylates GSK3β at Ser9 and inactivates its kinase activity, thus allowing the initiation of protein synthesis. Activation of the Akt/GSK3β pathway is observed in muscle *in vitro*, using anabolic stimulus on C2C12 myotubes. Administration of IGF-1 resulted in myotube hypertrophy, associated with hyperphosphorylation of both Akt and GSK3β (Rommel et al., 2001).

There are several regulatory points throughout the IGF-1/Akt/mTORC1 pathway, yet mTORC1 appears to be the gateway to downstream anabolic signaling. mTOR assembles into two distinct complexes, mTORC1 and mTORC2. mTORC1 consists of raptor (regulated associated protein of mTOR), mLST8, DEPTOR, PRAS40, and mTOR. mTORC2 consists of rictor (rapamycin insensitive companion of mTOR), mSIN1, mLST8, DEPTOR, and mTOR. mTORC1 activation is well described through PI3K/Akt signaling. Akt can phosphorylate several proteins that regulate mTORC1 activity including mTORC1 itself, PRAS40 and tuberous sclerosis complex 2 (TSC2) (Sancak et al., 2007; Vander Haar et al., 2007). Currently, the signaling mechanism for Akt through TSC2 is the most well described pathway. Akt phosphorylates TSC2 on multiple residues leading to its inactivation. TSC2 is a GTPase activating protein for Rheb. Therefore, inactivation of TSC2 by Akt increases the amount of GTP:Rheb complex bound to mTOR and leads to its activation. The second mechanism by which Akt can activate mTORC1 is through phosphorylation of the mTORC1 inhibitor PRAS40. Phosphorylated PRAS40 will disassociate from mTORC1, release its inhibition and increase mTOR kinase activity (Wang et al., 2012). In relation to mTORC1, there is a limited understanding of the role of mTORC2 in muscle protein synthesis and growth (Bentzinger et al., 2008). Initial studies have shown mTORC2 to be involved in organization of actin cytoskeleton and possibly phosphorylate Akt at Ser473 (Jacinto et al., 2004). In addition, there may be a coordinated effort for both mTOR complexes to work together for maximizing muscle protein synthesis under anabolic conditions (Ogasawara et al., 2020).

## The mTORC1 Complex

The mTORC1 complex has several related protein-protein complexes which regulate signaling activity. Each protein has a unique function in the complex. Raptor acts as a scaffolding protein to recruit downstream targets of mTOR, p70S6K, and 4E-BP1 (Hara et al., 2002; Kim et al., 2002). It is also the anchoring protein used by the Rag GTPases to recruit mTORC1 to the lysosome (Sancak et al., 2008). In skeletal muscle, raptor KO mice have a marked reduction in phosphorylation of both p70S6K and 4E-BP1 (Bentzinger et al., 2008). In addition, p70S6K and 4E-BP1 proteins have common mTORC1 signaling (TOS) motifs, which are essential for mTORC1-targeted phosphorylation (Dunlop et al., 2009). The raptor protein can also be modified at multiple phosphorylation sites. Phosphorylation of raptor appears to happen in a sequential manner (Foster et al., 2010). Once activated, mTOR will phosphorylate raptor at Ser^863^, which primes raptor for phosphorylation at several other sites including Ser^859/855^ (Foster et al., 2010). The phosphorylation events must be in the presence of adequate amino acid concentrations. In HEK293 cells, insulin-stimulated phosphorylation of raptor was not evident in amino acid depleted serum showing amino acid availability is critical for mTORC1 activity despite the availability of other growth factors (Foster et al., 2010). Raptor can also be phosphorylated by the 5′-adenosine monophosphate-activated protein kinase (AMPK) at Ser792, which inhibits raptor function and related mTORC1 activity (Gwinn et al., 2008). Skeletal muscle raptor phosphorylation at the AMPK targeted Ser792 is associated with body weight loss during cancer-associated muscle wasting (White et al., 2011).

The protein PRAS40 is another member of the TORC1 complex. PRAS40 has been shown to be an inhibitor of mTOR activity (Sancak et al., 2007; Wang et al., 2007). PRAS40 is bound to the inactive mTORC1 complex and directly inhibits substrate binding to raptor preventing downstream phosphorylation (Wang et al., 2007). Akt has been shown to phosphorylate and inhibit PRAS40 binding to raptor (Vander Haar et al., 2007), however, mTOR can phosphorylate PRAS40 as well (Foster et al., 2010). Upon activation from insulin or amino acids, activated mTOR can phosphorylate PRAS40 which facilitated its disassociation from the complex (Foster et al., 2010). Once PRAS40 is off the complex, raptor can bind p70S6K and 4E-BP1 for eventual phosphorylation. In male mice, muscle PRAS40 phosphorylation is responsive to circulating testosterone and muscle mass (White et al., 2012). Castration decreases phospho PRAS40, which is rescued with androgen add-back (White et al., 2012).

DEP domain-containing mTOR-interacting protein is a relatively recent addition to the mTORC1 complex, having an inhibitory function on mTORC1 activity (Peterson et al., 2009). mTORC1 and DEPTOR negatively regulate each other, depending on nutrient availability. In a low nutrient state, the PZD domain of DEPTOR binds to the C-terminal portion of mTOR and inhibits downstream signaling to p70S6K and 4E-BP1. In addition, it also activates mTORC2/Akt signaling by relieving the mTORC1 negative feedback inhibition of PI3K (Peterson et al., 2009). During nutrient availably and subsequent mTORC1 activity, DEPTOR is phosphorylated and released from the mTORC1 complex. The reduction in protein expression is also accompanied with a suppression of DEPTOR mRNA expression (Peterson et al., 2009). In C2C12 myotubes, the knockdown of DEPTOR increased protein synthesis and associated mTORC1 signaling (Kazi et al., 2011). DEPTOR knockdown, *in vivo*, resulted in an attenuation of immobilization-induced muscle atrophy associated with increased muscle protein synthesis (Kazi et al., 2011). The sensitivity of DEPTOR to atrophy conditions has been replicated by others showing DEPTOR expression increases with limb immobilization (Shimkus et al., 2018) and hind limb unloading (Roberson et al., 2020). The regulation of DEPTOR from nutrient availability does not appear as strong as mechanical loading, as fasting/refeeding did not alter DEPTOR protein expression (Shimkus et al., 2018). However, further investigation is needed to determine how DEPTOR is regulated under different nutrients and availability of amino acids.

## Negative Regulators of mTORC1

**FIGURE 3:**
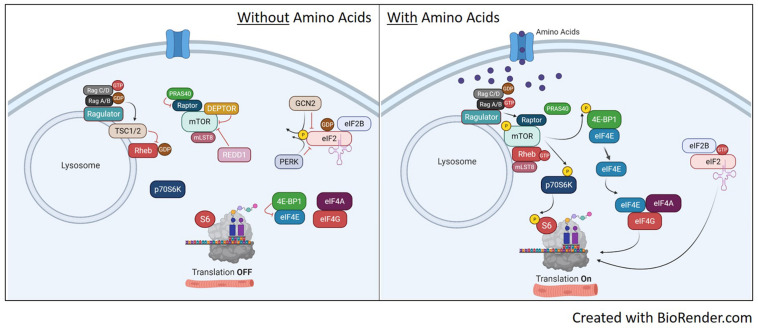
Translation pathways with or without amino acid availability. Without amino acid availability (left image), the heterodimeric Rag protein is loaded with GTP on Rag C/D while Rag A/B has the GDP. This conformation localizes the TSC1/2 complex to the lysosome to prevent GDP-Rheb conversion to GTP and prevents the recruitment of mTORC1 to the lysosome. This renders both S6 and 4E-BP1 unphosphorylated, shutting off translation. In addition, the inactive mTORC1 with be further suppressed by inhibitors DEPTOR, REDD1 and PRAS. On the eIF2 pathway, the absence of amino acids maintains GCN2 and PERK activity, which phosphorylates eIF2, inhibiting guanine nucleotide exchange of eIF2 by eIF2B. With eIF2 bound to GDP, it will release from the ribosome and stop translation. Upon availability of amino acids (right image), RagA/B is now loaded with GTP as it maintains binding to ragulator. This initiates removal of TSC1/2 from the lysosome and Rag-induced recruitment of mTORC1 to the lysosome via raptor. Once at the lysosome, mTOR kinase activity is activated by GTP-bound Rheb and will phosphorylate p70S6K and 4E-BP1 to initiate translation. mTORC1 activation will also disassociate inhibitors DEPTOR and PRAS40. Once dissociated, DEPTROR is quickly degraded. In relation to eIF2, the availability of amino acids will inhibit GCN2 and PERK, reversing phosphorylation on eIF2 and allowing guanine nucleotide exchange of eIF2 back to GTP. This will bind the eIF2 complex to the 40S ribosomal subunit and initiate protein translation.

There are several negative regulators of mTORC1 activity existing outside the mTORC1 complex. Two well documented inhibitors are AMPK and the protein regulated in DNA damage and development 1 (REDD1, also referred to as Rtp801 and DDIT4). Their respective interactions with the Akt/mTOR pathways are shown in Figures 2, 3. AMPK and mTOR are key energy sensors in the cell, and function to regulate processes to either inhibit or enhance ATP production depending on nutrient availability. AMPK will be discussed in more detail later in this review. REDD1 is thought to inhibit mTORC1 signaling through activation of upstream TSC2 (Brugarolas et al., 2004; Reiling and Hafen, 2004; Sofer et al., 2005). In addition, REDD1 protein and mRNA expression are increased with cellular stress events including ATP depletion (Sofer et al., 2005), DNA damage (Lin et al., 2005), endoplasmic reticulum stress (Wang et al., 2003; Protiva et al., 2008), and hypoxia (Shoshani et al., 2002; Brugarolas et al., 2004; Reiling and Hafen, 2004; Schwarzer et al., 2005). Furthermore, treatment with the synthetic glucocorticoid dexamethasone has shown to increase REDD1 mRNA and protein in skeletal muscle as well as L6 myotubes (Wang et al., 2006). Glucocorticoids such as cortisone are elevated during fasting states which, in part through REDD1, may play a role in the inhibition of mTORC1 signaling and the subsequent reduction in protein synthesis. REDD1 protein and mRNA expression was increased with 18 h of starvation in rats which coincided with a reduction in mTORC1 signaling (McGhee et al., 2009). Upon refeeding, REDD1 protein and mRNA expression was returned to baseline and mTORC1 signaling was increased. Interestingly, fasting-induced glucocorticoid concentrations directly correlated with REDD1 expression showing evidence of cross talk between energy-sensitive hormones and energy-sensitive signaling within muscle (McGhee et al., 2009). Finally, the loss of REDD1 during a mechanical-overload hypertrophy stimuli enhanced the rate of muscle protein synthesis (Gordon et al., 2016), again, suggesting its role as a negative regulator of muscle protein anabolism.

## AMPK Promotes Catabolism Over Anabolism

AMPK is activated through the buildup of low energy phosphate group, AMP or by phosphorylation by one or more upstream kinases at a threonine residue within the activation loop of the α subunit (Hawley et al., 1996; Stein et al., 2000). The multiple targets that AMP can activate will induce a large activation in the activity of AMPK with relatively small changes in AMP. The energy state of the cell is not solely monitored by AMP concentrations. High ATP concentrations will oppose activation of AMP-induced pathways. Thus, the AMP:ATP ratio appears to the critical readout of cellular energy status and regulator of AMPK activity.

During physiological conditions, AMPK can be regulated by chemical mediators of metabolism in addition to ATP:AMP levels. Cellular levels of phosphocreatine can allosterically inhibit AMPK activity (Ponticos et al., 1998). In addition, glycogen content of the cell can affect AMPK activity (Hudson et al., 2003; Polekhina et al., 2003). The β-subunits of AMPK contain a glycogen binding domain. Reports in human and rodent muscle show high glycogen stores can inhibit AMPK activation (Wojtaszewski et al., 2002, 2003). Over expression of AMPK in culture has shown AMPK to localize in large glycogen granules (Hudson et al., 2003). Glycogen will not only bind AMPK, but also contain in close proximity glycogen synthase, a substrate of AMPK. Considering AMPK is allosterically regulated by phosphocreatine and glycogen stores, it has been speculated that AMPK is regulated by both short and long term energy stores (Hardie, 2003).

## AMPK Targets mTORC1 to Suppress Protein Synthesis

AMPK has been shown to inhibit protein synthesis in skeletal muscle (Rolfe and Brown, 1997; Bolster et al., 2002; Deshmukh et al., 2008; Thomson et al., 2008; Mounier et al., 2009), liver (Reiter et al., 2005), and cultured myotubes (Williamson et al., 2006; Tong et al., 2009). The potency of AMPK signaling was described by Pruznak et al. (2008), showing AMPK activation can override the stimulatory effects of leucine on muscle protein synthesis (Pruznak et al., 2008). In contrast, deletion of the AMPKα1 gene in primary myotubes resulted in cell hypertrophy (Mounier et al., 2009). The mechanism by which AMPK inhibits muscle protein synthesis is through the inhibition of the mTORC1 complex (Bolster et al., 2002; Horman et al., 2002; Chan et al., 2004; Gwinn et al., 2008). There are currently three proposed mechanisms by which AMPK can inhibit mTORC1 signaling. The first is through phosphorylation of mTOR on Thr2446 (Cheng et al., 2004). This process does not directly inhibit mTOR activity, however, phosphorylation at Ser2446 prevents phosphorylation of Ser2448 which has been shown to increase mTOR activity (Bolster et al., 2002; Chiang and Abraham, 2005). The second method, and perhaps the best described mechanism, is through AMPK-mediated phosphorylation of the tuberous sclerosis complex 2 (TSC2) gene product Tuberin on Thr1227 and Ser1345 (Inoki et al., 2003). TSC2 combines with TSC1 to form a GTPase activator protein (GAP) for the Ras homolog enriched in brain (Rheb), causing an increase in GDP bound to Rheb (Zhang et al., 2003; Long et al., 2005a). The binding of the GDP:Rheb complex to mTORC1 inhibitors mTOR. The third mechanism, as discussed earlier in the review, is the phosphorylation of raptor (Gwinn et al., 2008). This promotes binding of the 14-3-3 protein and inhibition of raptor to signal downstream to p70S6K and 4E-BP1.

The AMPK pathways has been heavily investigated in muscle in regards to other aspects of mTORC1 signaling. In C2C12 cells, AICAR-induced AMPK activation showed a reduction in protein synthesis, polysome aggregation and downstream mTORC1 signaling proteins 4E-BP1, p70S6K and eEF2 (Williamson et al., 2006). Although Akt, upstream of mTORC1, remained unaffected with AICAR treatment, downstream AMPK targets raptor and TSC2 were effected with AMPK activation. In addition, AICAR increased the amount of TSC1 bound to TSC2 (Williamson et al., 2006), suggesting increased GTPase activity and inhibition of Rheb. A study by Du et al. (Tong et al., 2009) treated C2C12 cells with both AICAR and IGF-1 to determine if AMPK signaling can inhibit IGF-1 signaling, with the hypothesis that IGF-1 signaling originates upstream of mTORC1. AICAR treatment without IGF-1 resulted in cell atrophy caused by a reduction in signaling related to protein synthesis and an increase in markers of protein degradation. The addition of IGF-1 did not rescue the inhibition of AICAR treatment despite a significant increase in Akt, supporting the proposed mechanism of potent mTORC1 inhibition by AMPK.

AMPK has been examined in rodent models of muscle hypertrophy to determine its role in growth suppression. Overload-induced hypertrophy of the plantaris muscle was enhanced in AMPKα1−/− mice. This occurred in conjunction with an increase in phosphorylation of downstream targets of mTORC1 signaling p70S6K and 4E-BP1 (Mounier et al., 2009). In contrast, AICAR treatment resulted in a reduction in the percentage of plantaris muscle mass gained after 1 week of over load (Gordon et al., 2008). In the same study, there was a significant negative correlation between the percentage of plantaris hypertrophy and AMPK phosphorylation status in the plantaris muscle. In addition, there were also negative correlations between phosphorylation status of AMPK and p70S6K, eEF2 and 4E-BP1.

Catabolic signaling through AMPK can override amino acid-induced mTORC1 activation. AICAR treatment prevented leucine-stimulated protein synthesis in the mouse gastrocnemius muscle (Pruznak et al., 2008). The prevention of synthesis was accompanied with the prevention of mTOR activation. AMPK can phosphorylate and activate TSC-2, which subsequently inactivates mTOR. AICAR treatment did not increase the phosphorylation of TSC-2 or alter TSC-1/TSC-2 binding. However, AICAR treatment did increase phosphorylation of raptor independent of leucine treatment. The activation of downstream signaling proteins p70S6K1, 4E-BP1, and eIF4F were increased with leucine administration and prevented when leucine was given with AICAR treatment. This data is in agreement with the results from Du et al. (Du et al., 2007) who showed AICAR treatment prevents a leucine-induced increase in protein synthesis in C2C12 myoblasts. In support of these data, myoblasts expressing a dominant negative AMPKα subunit were administered AICAR. Without AMPK activation, leucine was able to increase protein synthesis even with AICAR treatment. Once again, suggesting AMPK-induced inhibition of protein synthesis was through the reduction in mTORC1. These results support the hypothesis that cellular energy demands can supersede the anabolic potential of amino acid availability.

## Amino Acid Availability and mTORC1 Activity

Although mTORC1 is a key component of the IGF-1/insulin signaling pathway, mTORC1 can be activated independent of upstream signaling by amino acids (Potier et al., 2009). Branched-chain amino acids (BCAAs), especially leucine, are potent regulators of mTORC1 activity and increase rates of protein synthesis (Goberdhan et al., 2009). Infusion of an amino acid mixture into resting human subjects increased protein synthesis as early as 30 min after infusion and remained elevated for 90 min (Bohe et al., 2001). Amino acid infusion has been shown to increase phosphorylation of downstream targets of mTORC1, p70S6K, and 4E-BP1 (Greiwe et al., 2001; Liu et al., 2001) without effecting Akt (Greiwe et al., 2001) or its downstream target GSK3β (Liu et al., 2004). In the rodent, mTORC1 activity is necessary for BCAAs to induce anabolic signaling, as rapamycin prevented leucine-induced increased phosphorylation of p70S6K and 4E-BP1 (Anthony et al., 2000).

Despite strong evidence suggesting BCAAs activate mTORC1 signaling, the direct mechanism for mTORC1 activation remains unclear, especially in skeletal muscle. It has been proposed that BCAAs work through both a TSC1/2 dependent (Gao et al., 2002) and independent (Smith et al., 2005) manner to activate mTORC1 activity. The TSC1/2 complex can mediate amino acid signaling to mTORC1 (Gao et al., 2002), which could be regulated through relative localization of TSC1/2 and mTOR on the lysosome (Demetriades et al., 2014; Menon et al., 2014). Other reports have shown evidence for TSC1/2 independent activation of mTORC1 signaling through RAG and Rheb. In mammals, there are four RAG GTPases (A-D) shown to have a role in amino acid signaling to mTORC1 (Schurmann et al., 1995; Kim et al., 2008; Sancak et al., 2008). Amino acids can convert RAG to an active formation including Rag A/B loaded with GTP and Rag C/D loaded with a GDP. The active Rag A/B recruits the mTORC1 complex to the lysosome through raptor where it is activated by Rheb (Sancak et al., 2008). Rheb activates mTORC1 by direct associated and activation of the mTOR catalytic domain (Long et al., 2005a,b). The regulation of mTORC1 activation with and without amino acid availability is illustrated in Figure 3.

## Regulation of Amino Acid Metabolism

Amino acid metabolism has been well described, especially in the context of insulin resistant and obesity (White and Newgard, 2019). In skeletal muscle, the balance between amino acid catabolism and anabolism is complex, due to both metabolic and anabolic flux of the myofiber. BCAAs, in particular, are a widely used source to generate TCA/Krebs cycle intermediates for energy production. Although the majority of amino acid metabolism occurs in the liver, skeletal muscle has high expression of the branched-chain aminotransferase (BCAT). Interestingly, despite its metabolic nature, the liver does not express BCAT, rendering a unique pathway of BCAA metabolism to skeletal muscle (Hutson, 1989; White et al., 2020). BCAT-mediated transamination of leucine generates α-ketoisocaproate, the first step of BCAA catabolism in muscle. The second step of leucine catabolism is an irreversible oxidative decarboxylation of α-ketoisocaproate, which is catalyzed by the branched-chain α-keto acid dehydrogenase (BCKDH). This reaction is a rate-limiting step in leucine metabolism (Harris et al., 1990, 1994).

**FIGURE 4:**
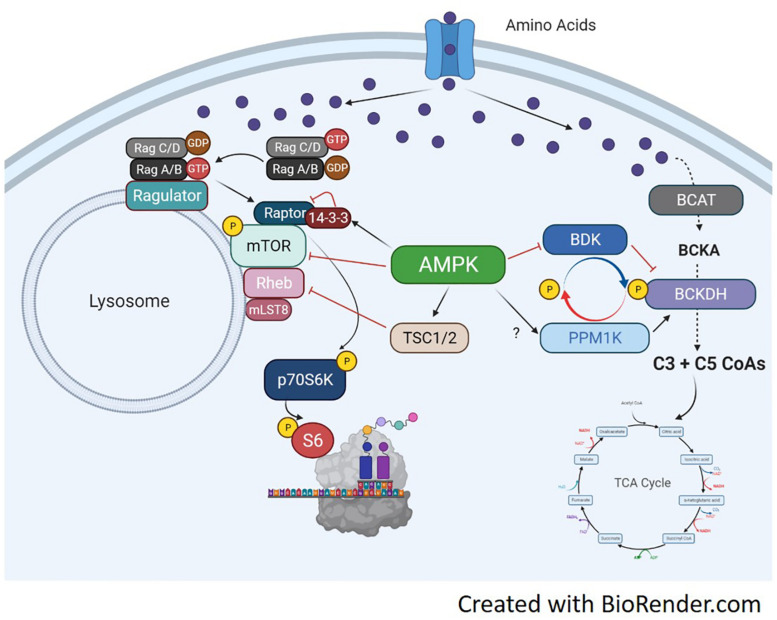
Molecular mechanisms of amino acid trafficking. Amino acids, especially BCAAs, enter the cell via their respective transporters. Once in the cell, depending on metabolic need, they can be metabolized for energy or used for other biochemical processes like protein synthesis. If needed for energy, the enzyme BCAT metabolizes the BCAA into branched-chain keto acids, which undergo a series of catabolic reactions to produce C3 and C5 CoAs by BCKDH. The C3/5 CoAs are then converted to either acetyl CoA or Succinyl CoA and can enter the TCA cycle. BCKDH is regulated by phosphorylation/dephosphorylation by the kinase BDK and the phosphatase PPM1K, respectively. Phosphorylation of BCKDH by BDK inhibits BCKDH activity while dephosphorylation by PPM1K activates BCKDH and increases generation of BCAA-derived CoAs for energy production. The activation of the BCAT/BCKDH pathway pulls amino acids away from the associated mTORC1 complexes and inhibits protein synthesis. If BDK is able to phosphorylate BCKDH, or PPM1K is inhibited, amino acids would be available to active mTORC1 and initiate protein translation. AMPK can regulate BCAA metabolism by increasing PPM1K and lowering BDK expression, which will activate BCKDH and increase BCAA flux to the TCA cycle. AMPK can simultaneously block mTORC1 signaling through activation of TSC1/2 or direct inhibition of mTOR and raptor.

Branched-chain α-keto acid dehydrogenase is a highly regulated dehydrogenase enzyme responsible for metabolizing branched-chain keto acids (BCKA) into branched-chain acyl CoAs. The branched-chain CoAs are further metabolized into acetyl CoA or Succinyl CoA and used as TCA intermediates for energy (Walejko et al., 2021). The multi-subunit BCKDH complex consists of three components including the e1, e2, and e3 subunits. Each subunit carries out different reactions to convert BCKAs into branched-chain acyl CoAs. There are two opposing regulators of BCKDH activity, the BCKDH kinase (BDK) and the PPM1K phosphatase (also referred to as PP2Cm) (White et al., 2018). Both enzymes perform phosphorylation and dephosphorylation, respectively, of serine 293 of the e1a subunit. In the liver, increased phosphorylation of BCKDH on serine 293 occurs secondary to elevated expression of BDK, and decreased expression of PPM1K (She et al., 2007; Lian et al., 2015). Murine knockout models of either BDK or PPM1K (Joshi et al., 2006; Lu et al., 2009) show the significance of their respective role in BCAA metabolism, as each manipulation effects circulating BCAAs accordingly. Currently, there is limited understanding of this pathway in skeletal muscle, especially in regards to anabolic and catabolic conditions. BCAA fate and the antagonist relationship between BCKDH and PPM1K is shown in Figure 4.

As AMPK is a potent inhibitor of mTORC1 activity, it would also make sense that AMPK would regulate muscle amino acid metabolism. AICAR-induced AMPK activation can increase BCKDH activity in skeletal muscle through a reduction in BDK protein (Lian et al., 2015). Although AICAR increased PPM1K in liver and adipose tissue, muscle PPM1K was not effected by AICAR treatment (Lian et al., 2015). Skeletal muscle PGC-1α over-expression increases gene expression of branched-chain amino transferase (BCAT) 2 and BCKDH, while BDK was not changed. BCAAs levels in the PGC-1α mice were decreased in both muscle and blood (Hatazawa et al., 2014). These outcomes show the coupled relationship between two potent metabolic regulators, i.e., AMPK and PGC-1α, and the shift from anabolism to BCAA catabolism.

The extent of BCAA metabolism can have an impact on global muscle metabolism, as excess BCAAs or branched-chain ketoacids can inhibit insulin signaling in muscle *in vitro* (Moghei et al., 2016; Biswas et al., 2020) and *in vivo* (White et al., 2016, 2018; Wang et al., 2019). Interestingly, this result is dependent on certain BCAAs or a mixture of BCAAs as valine does seem to interfere with myotube insulin signaling *in vitro* (Rivera et al., 2020). Nutrient availability may also regulate metabolic fate of leucine. In C2C12 myotubes, leucine is used preferentially for protein synthesis rather than oxidation for energy production (Estrada-Alcalde et al., 2017). However, in the setting of high palmate, leucine oxidation increases and its incorporation into proteins decreases (Estrada-Alcalde et al., 2017). Moreover, high fat feeding increase BCKDH activity in muscle promoting amino acid catabolism (White et al., 2016). This again, points to the complexity of muscle metabolism and substrate availability altering BCAA trafficking. The proposed BCAA trafficking and related AMPK signaling pathways are shown in Figure 4.

In the context of muscle mass regulation, muscle BDK knockout mice have no overt muscle mass phenotype under a typical chow diet, despite a lower BCAA concentration in blood and muscle (Ishikawa et al., 2017). However, under a low protein diet, the lack of BDK magnifies myofibrillar protein loss associated with a reduction in mTORC1 signaling activity. Of note, protein restriction resulted in a reduction of myofibrillar protein synthesis, but not total (soluble) protein, indicating a preferential degradation of myofibrillar proteins to compensate for the low protein diet. A natural hypothesis would point to autophagy as a mechanism to provide amino acids during the restricted feeding. However, this study showed no difference in LC3I/II protein expression, and so the authors concluded autophagy was not playing a role and contributing to the myofibrillar degradation during the low protein diet (Ishikawa et al., 2017). This study highlights the interaction between BCAA metabolism and protein synthesis pathways, supporting an interactive crosstalk between the two processes. More work is needed to gain a better understanding of the molecular network between BCAA trafficking and mTORC1 signaling.

## Conclusion and Perspective

Together, muscle protein synthesis is an interactive process, taking input from numerous anabolic and catabolic pathways. The unique plasticity of skeletal muscle adds more layers of regulation, incorporating both metabolic demands and mechanical stimuli into these already intricate pathways. Moreover, there must be an adequate combination of mechanical stimuli and nutritional availability to maintain or hypertrophy myofiber size. In relation to other tissues, especially tumor biology, the molecular mechanisms involved in skeletal muscle protein synthesis are less developed. This concept is supported by the fact that the majority of citations in this review investigating amino acid metabolism and regulation of mTORC1 are not in muscle tissue. This is interesting, considering the extensive protein content of skeletal muscle and the potential utility of skeletal muscle as a model to investigate the complexities of protein synthesis. In regards to muscle signaling pathways, the IGF-1/insulin pathway dominates the literature since muscle is a major therapeutic target for diabetes drugs. However, the anabolic pathways in muscle have been relatively neglected since muscle atrophy/wasting is still considered a secondary symptom among various diseases and not targeted as commonly for pharmaceutical intervention.

A better understanding of the interface between muscle amino acid metabolism and synthesis pathways could uncover additional regulators of muscle protein synthesis. The high expression of BCAT in skeletal muscle supports the preference of branched-chain amino acids as a bioenergetic substrate. There is a gap in our understanding of fate decisions of BCAAs and anabolic signaling in muscle. This could be a result of the temporal nature of muscle energetics, having diverse metabolism with changes in nutrient availability and contractile activity. AMPK is an obvious regulator of BCAA fate, effecting both catabolic and anabolic signaling pathways, including direct/indirect effects on mTORC1 and BCKDH. However, it would not be surprising to identify additional regulators of BCAA metabolism having an impact on both catabolic and anabolic processes.

As for translational potential of these pathways to offset muscle atrophy/wasting, identification of anabolic pathways, unique to skeletal muscle, could open up therapeutic targets. The potency of these pathways to regulate muscle mass is supported by strong *in vivo* studies using preclinical models discussed throughout his review. Manipulation of key regulatory proteins within the mTORC1 signaling pathway can accelerate (Ishikawa et al., 2017) or preserve (Kazi et al., 2011) the loss in muscle mass and/or protein synthesis under atrophy-promoting conditions. Since we now have a general understanding of these pathways, why are there no available drugs to offset muscle wasting? The challenge is to identify key targets within these complex pathways and manipulate them in a muscle-specific manner. The mTORC1 pathway is tightly controlled and ubiquitous across many cell types. Promoting muscle anabolism by manipulating global mTORC1 activity will most likely alter the delicate balance of non-muscle cells and promote unchecked growth and malignancies. Finding key regulators within the mTORC1 pathway, specific to muscle would be ideal for drug development. This warrants continued investigation of anabolic pathways, especially within skeletal muscle.

## Author Contributions

The author confirms being the sole contributor of this work and has approved it for publication.

## Conflict of Interest

The author declares that the research was conducted in the absence of any commercial or financial relationships that could be construed as a potential conflict of interest.

## Abbreviations

4E-BP1: the eukaryotic initiation factor 4E binding protein 1
ADP: Adenine diphosphate
AICAR: 5-Aminoimidazole-4-carboxamide ribonucleotide
Akt: AKR thymoma
AMP: Adenine monophosphate
AMPK: AMP-activated kinase
ATP: Adenine triphosphate
BCAAs: Branched-chain amino acids
BCAT: Branched-chain amino acid aminotransferase
BCKDH: branched-chain α-keto acid dehydrogenase
BDK: BCKDH kinase
BKAs: branched-chain keto acids
DEPTOR: DEP domain-containing mTOR-interacting protein
eEF: Eukaryotic elongation factor
eIF: eukaryotic initiation factor
GAP: GTPase-activating protein
GCN2: general control non-derepressible 2
GDP: Guanosine diphosphate
GEF: guanine nucleotide exchange factor
GSK3β: glycogen synthase kinase-3
GTP: Guanosine-5 ′ -triphosphate
HRI: heme-regulated inhibitor kinase
IGF-1: Insulin-like growth factor
IRS: insulin receptor substrate
mLst8: mammalian LST8 homolog
mSin1: the mammalian stress-activated protein kinase-interacting 1
mTOR: mechanistic Target of Rapamycin
mTORC1: mammalian/mechanistic Target of Rapamycin Complex 1
mTORC2: mammalian/mechanistic Target of Rapamycin Complex 2
P70S6K: P70 Ribosomal protein S6 kinase
PDK-1: phosphoinositide-dependent kinase 1
PERK: eukaryotic translation initiation factor 2-alpha kinase 3
PGC-1α: Peroxisome proliferator-activated receptor gamma coactivator 1-alpha
PI3K: phosphatidylinositol 3-kinase
PI3P: phosphatidylinositol 3-phosphate
PIP3: phosphatidylinositol (3,4,5)-trisphosphate
PKB: Protein kinase B
PKR: double stranded RNA-dependent protein kinase
PPM1K: protein phosphatase 1K
PRAS40, proline-rich Akt substrate: 40 kDa
Rag: Ras related GTP binding
Raptor: the regulatory-associated protein of mTOR (Raptor)
REDD1: protein regulated in DNA damage and development 1
Rheb: Ras homolog enriched in brain
Rictor: the rapamycin-insensitive companion of mTOR
RTK: receptor tyrosine kinase
TCA: tricarboxylic acid
TOR: Target of Rapamycin
TSC: Tuberous sclerosis complex.
